# Prediction of the binding mechanism of a selective DNA methyltransferase 3A inhibitor by molecular simulation

**DOI:** 10.1038/s41598-024-64236-9

**Published:** 2024-06-12

**Authors:** Genki Kudo, Takumi Hirao, Ryuhei Harada, Takatsugu Hirokawa, Yasuteru Shigeta, Ryunosuke Yoshino

**Affiliations:** 1 Physics Department, Graduate School of Pure and Applied Sciences, University of Tsukuba, 1-1-1 Tennodai, Tsukuba, Ibaraki 305-8571 Japan; 2 Doctoral Program in Medical Sciences, Graduate School of Comprehensive Human Sciences, University of Tsukuba, Tsukuba, Ibaraki 305-8575 Japan; 3 Division of Biomedical Science, Faculty of Medicine, University of Tsukuba, 1-1-1 Tennodai, Tsukuba, Ibaraki 305-8575 Japan; 4 Center for Computational Sciences, University of Tsukuba, 1-1-1 Tennodai, Tsukuba, Ibaraki 305-8577 Japan; 5 Transborder Medical Research Center, University of Tsukuba, 1-1-1 Tennodai, Tsukuba, Ibaraki 305-8575 Japan

**Keywords:** DNA methyltransferase, DNMT3A, Molecular dynamics simulation, Protein inhibitor, Selective inhibitor, Drug discovery and development, Structure-based drug design, Computational biology and bioinformatics

## Abstract

DNA methylation is an epigenetic mechanism that introduces a methyl group at the C5 position of cytosine. This reaction is catalyzed by DNA methyltransferases (DNMTs) and is essential for the regulation of gene transcription. The DNMT1 and DNMT3A or -3B family proteins are known targets for the inhibition of DNA hypermethylation in cancer cells. A selective non-nucleoside DNMT3A inhibitor was developed that mimics S-adenosyl-l-methionine and deoxycytidine; however, the mechanism of selectivity is unclear because the inhibitor–protein complex structure determination is absent. Therefore, we performed docking and molecular dynamics simulations to predict the structure of the complex formed by the association between DNMT3A and the selective inhibitor. Our simulations, binding free energy decomposition analysis, structural isoform comparison, and residue scanning showed that Arg688 of DNMT3A is involved in the interaction with this inhibitor, as evidenced by its significant contribution to the binding free energy. The presence of Asn1192 at the corresponding residues in DNMT1 results in a loss of affinity for the inhibitor, suggesting that the interactions mediated by Arg688 in DNMT3A are essential for selectivity. Our findings can be applied in the design of DNMT-selective inhibitors and methylation-specific drug optimization procedures.

## Introduction

Epigenetic modifications through DNA methylation are essential for the regulation of genome structure and gene expression^1,2^. This process involves the addition of a methyl group at the C5 position of cytosine to produce 5-methylcytosine; nearly 80% of cytosines in CpG islands, which are composed of numerous CG sequences, undergo methylation^3,4^. The DNA methyltransferase (DNMT) family catalyzes DNA methylation using the methyl group provided by S-adenosyl-l-methionine^5^. Two families of DNMTs have been described: DNMT1 is responsible for the maintenance of methylation during DNA replication, and DNMT3A and DNMT3B perform de novo DNA methylation^6,7^. Hypermethylation of CpG islands in certain promoter sequences results in the inactivation of tumor suppressor genes and is associated with tumor progression^8,9^. As DNA hypermethylation is common in cancer cells, including excessive methylation of certain tumor suppressor genes, the suppression of DNA hypermethylation via the inhibition of DNMTs may be a useful strategy for the development of anticancer drugs^10–12^.

**Figure 1:**
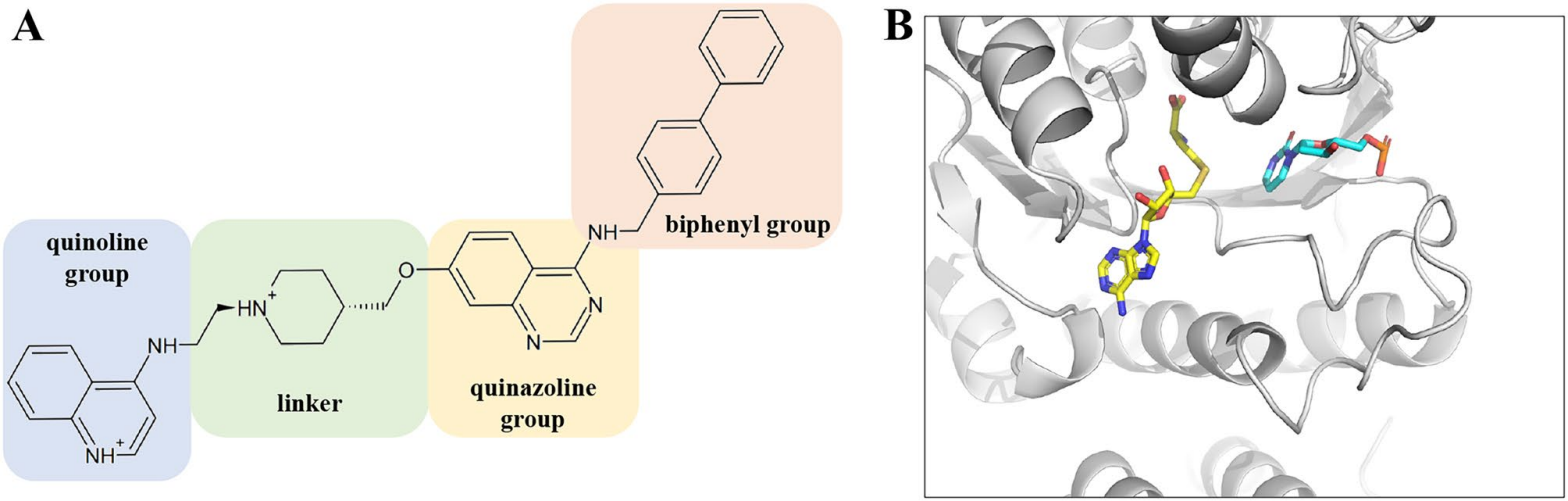
Chemical structure of 4-([1,1′-Biphenyl]-4-ylmethylamino)-7-((1-(2-(quinolin-4-ylamino)ethyl) piperidin-4-yl)methoxy) quinazoline and the substrates SAH and cytosine in DNMT3A. (**A**) Chemical Structure of the DNMT3A-selective inhibitor. Color boxes represent the constituents of fragment units. With LigPrep, the nitrogen in the linker and quinoline group is protonated. (**B**) Binding pose of SAH and cytosine in the X-ray structure (PDB ID: 6F57). SAH and cytosine are colored similarly to the mimic in the inhibitor (quinazoline and quinoline group, respectively).

The DNMT-targeting inhibitors, 5-azacytidine (azacitidine) and 5-aza-2ʹ-deoxycytidine (decitabine), are nucleoside analog FDA-approved inhibitors. However, their selectivity and bioavailability are limited^11^. Considering that drug selectivity is crucial in compound development to mitigate side effects^13,14^, several nucleoside analogs and non-nucleoside compounds with various scaffolds have been identified and assessed for their DNMT selectivity^15–18^. Achieving selectivity for each DNMT represents a significant challenge due to the structural conservation of active sites within the DNMT family (Fig. S1). Lamiable-Oulaidi F et al. synthesized a nucleoside analog inhibitor that does not inhibit DNMT3b at 100 μM, while exhibiting an IC_50_ value of 7.2 ± 2.2 μM against DNMT1^19^. Halby et al. designed a non-nucleotide mimetic compound (Fig. 1) based on the substrates S-adenosyl-l-methionine and deoxycytidine. This compound consists of four chemical entities—quinoline, quinazoline, connecting linker, and biphenyl groups and shows 100-fold selectivity (EC_50_) for DNMT3A compared to DNMT1 (DNMT3A: 1.1 ± 1.2 µM, DNMT1: 100 ± 3 µM)^20^. However, the mechanism of selectivity for each DNMT by these inhibitors is unclear.

Elucidation of complex structures provides insights into the binding mode, and identification of the mechanism of selectivity is crucial for rational design of DNMT family inhibitors. Computational methods, such as docking and molecular dynamics (MD) simulations, are also efficient tools for drug discovery and design^21–41^. Docking simulations can predict compound-binding poses and estimate the fit of a compound in the binding pocket of a target protein^27,28^. MD simulations are used to analyze the atomic-level dynamics of biopolymers in solution based on Newton’s equations of motion and can predict the function of proteins and stability of binding molecules^29–32^. Such MD simulations have been used in research on the DNMT family^33–38^. While docking poses have been predicted in published research, the validity of docking poses, binding free energies, and the contribution of interactions with amino acid residues can be evaluated by applying MD simulations to docking poses. Therefore, applying these simulation techniques to inhibitors will identify unique amino acid residue interactions required for selectivity and inform the design of various scaffolds^39–41^.

In the present study, we focused on a DNMT3A-selective inhibitor and performed docking and MD simulations to predict the structure of the selective inhibitor–DNMT3A complex. Using binding free energy calculations, structural comparisons of DNMT3A and DNMT1, and residue scanning calculations, we aimed to determine the key residue of DNMT3A responsible for the selectivity of the inhibitor.

## Methods

### Protein and ligand preparation

Protein and ligand three-dimensional (3D) structures were prepared as follows: The 3D structure of DNMT3A was accessed from the Protein Data Bank (PDB ID: 6F57)^42^. DNA and S-adenosyl-homocysteine were removed from the structure and missing residues in the PDB file were complemented using the protein preparation module in Maestro^43^. The structure of the selective inhibitor was previously published by Halby et al. (Fig. 1A)^20^. The 3D structure and ionized state were prepared using the LigPrep module in Maestro^43^. The appropriate ionization state of the inhibitor was generated at pH 7.0 with Epik (Fig. 1)^44^. The OPLS3e force field was used for protein and ligand preparation^45^.

### Docking simulation

The selective inhibitor was docked into the catalytic site of DNMT3A using DOCK 6.9^46^. We selected docking spheres within 6 Å of SAH and cytosine in the DNMT3A crystal structure, and the docking grid space was generated around 7 Å from these spheres with grid spacing set to 0.3 Å. With flexible ligand docking, Grid Score was set as the primary scoring to restrict the generated poses, and the Hawkins GB/SA Score was set as the secondary scoring to re-rank these poses with a high Grid Score. The top five poses with the best Hawkins GB/SA scores were used for the initial structure in the MD simulations.

### MD simulation

To perform the MD simulations, a system of initial structures from the docking simulation was prepared. For each docking conformation, the RESP charge of the selective inhibitor was calculated using the HF/6-31G in Gaussian 16^47^. The charge parameter was generated using the antechamber module in AmberTools^48^. The complex structure was placed in a 120 Å long cubic box, which was filled with water molecules. Cl^–^ ions were added to the box to neutralize the total charge of the system. The system was generated using the tLEaP module in the Amber biomolecular simulation programs^48^. FF14SB, General Amber Force Field (GAFF), and TIP3P were used as force field parameters for the protein, ligand, and water molecules, respectively^49–51^. The initial structures were subjected to energy minimization, NVT equilibration, and NPT equilibration. For energy minimization, every 200 steps were applied with and without positional restraints (10 kcal/mol/Å^2^) on the heavy solute atoms. After minimization, NVT equilibration with V-rescaling was performed at 300 K for 200 ps with position restraints (10 kcal/mol/Å^2^)^52^. NPT equilibration with a Berendsen barostat was performed at 300 K and 1 bar for 800 ps^53^. In NPT equilibration, position restraints were gradually reduced to 0 kcal/mol/Å^2^﻿. The constraint algorithm and time step were set to LINCS and 2 fs, respectively^54^. MD simulations after energy minimization were performed using GROMACS 2021.5^55^. We performed 200 ns MD simulations five times at different initial velocities under the NPT ensemble to relax the complex structure and assess the inflexibility and stability of each pose. The snapshot recording interval was set at 100 ps. The trajectories were fitted to the C_α_ atom in the initial step of the production run for each run.

To identify the most inflexible and stable docking pose, we calculated the root-mean-square deviation (RMSD) and binding free energy using the molecular mechanics Poisson-Boltzmann surface area (MM/PBSA) method^56^. The reference structure for the RMSD calculation was used as the initial structure of the 200 ns production run. The RMSD of the heavy atoms of the inhibitor was calculated by superimposing the C_α_ atoms in the protein. For the trajectories of complex structures with an inflexible binding pose, the binding free energy (Δ*G*_bind_) was calculated using gmx_MMPBSA^57^ by summing the four terms: Δ*E*_bonded_, Δ*E*_nonbonded_, Δ*G*_polar_, and Δ*G*_nonpolar_. Each term represents the bonded energy, nonbonded energy such as electrostatic and van der Waals, solvation energy for Poisson − Boltzmann models, and nonpolar constituent modeled as linearly proportional to the solvent-accessible surface area (SASA). The binding free energy was calculated using 100 snapshots recorded during the last 100 ns of each trajectory. The most inflexible and stable binding pose was predicted based on the RMSD and binding free energy results.

We identified the key residue responsible for selectivity between DNMT3A and the inhibitor in several stages. First, we analyzed the energy decomposition of residues within 6 Å of the selective inhibitor, based on the binding free energy results. Subsequently, we performed a structural alignment of DNMT3A and DNMT1 to assess the presence of different side chains for the residues with a high contribution to binding. We performed residue scanning calculations using BioLuminate for residues with a different side chain in the corresponding position in DNMT1^58^. The representative structure for residue scanning was selected from the clusters of the inhibitor’s conformation recorded in the last 100 ns of five MD simulation runs. Clustering was performed using the GROMOS method in GROMACS. In the representative structure, amino acid mutations with overlapping positions in DNMT1 were added to the candidate residues responsible for inhibitor selectivity. Δ*G*_bind_ for the selective inhibitor was performed using Prime MM-GBSA^59^, and the difference between post- and pre-mutation Δ*G*_bind_ was calculated as ΔΔ*G*_bind_.

## Results and discussion

### Docking simulation

**Figure 2:**
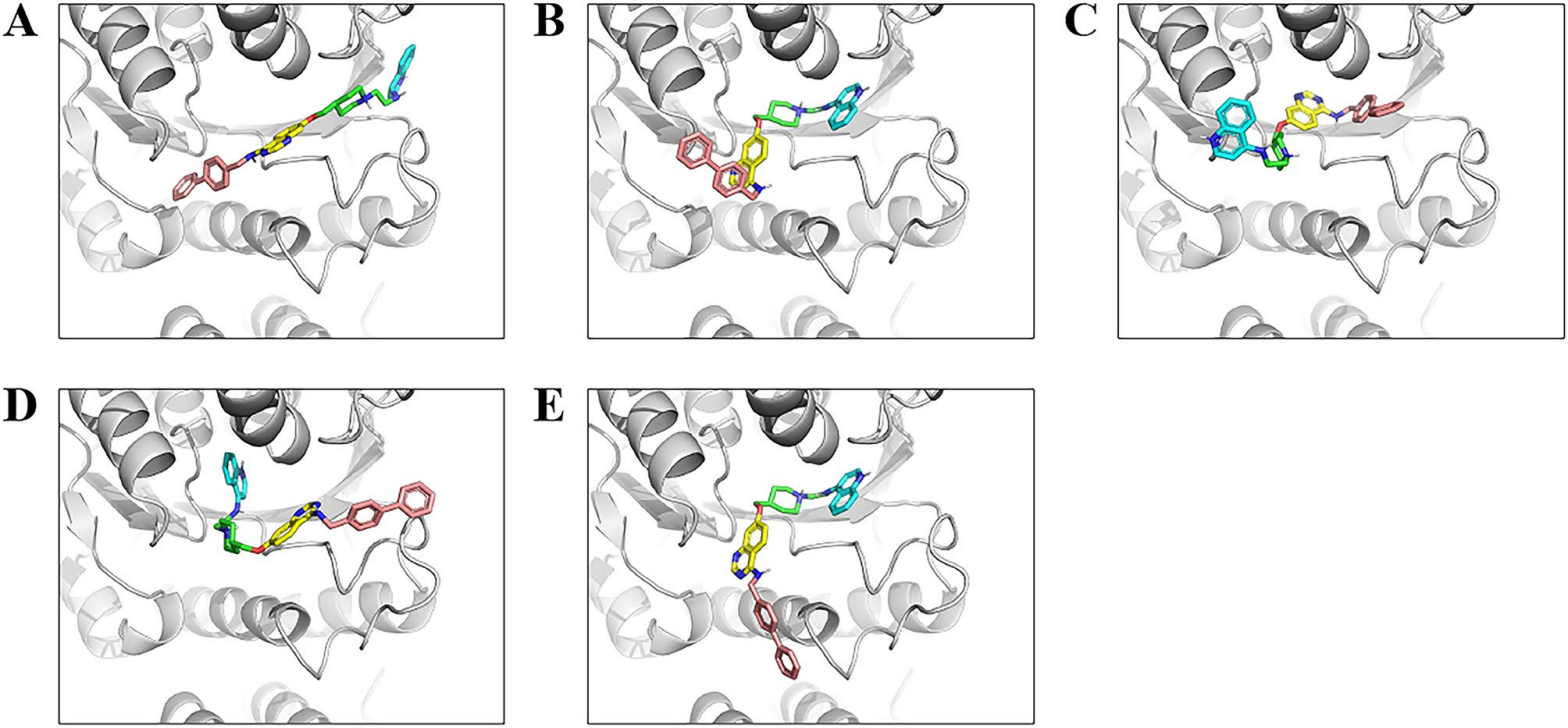
Top five docking poses based on Hawkins GB/SA scoring. (**A**)–(**E**) The four chemical groups of the inhibitor in the model are indicated by different colors (see Fig. 1).

The five complex structures according to the Hawkins GB/SA Score are shown in Fig. 2A–E and Table 1. No. 1 has a score of – 58.16 kcal/mol, a difference of more than 5 kcal/mol compared to the other poses. Although the score difference between Nos. 2, 3, 4, and 5 was less than 1.0 kcal/mol, the positions of the four groups in the selective inhibitor were at different locations. Figure 1B shows the binding pose of SAH and cytosine in the X-ray structure (PDB ID: 6F57). All docking poses were placed around the SAH and cytosine positions, indicating that these positions covered the catalytic site in DNMT3A. In Nos. 2 and 5 of the docking poses, the quinazoline and quinoline groups were positioned along the SAH and cytosine positions, respectively, conforming to the compound design concept proposed by Halby et al.^20^.

**Table 1:** Hawkins GB/SA score of top five docking poses.

Rank	Hawkins GB/SA score (kcal/mol)
No. 1	– 58.16
No. 2	– 53.15
No. 3	– 52.39
No. 4	– 52.25
No. 5	– 52.23

### MD simulation

**Figure 3:**
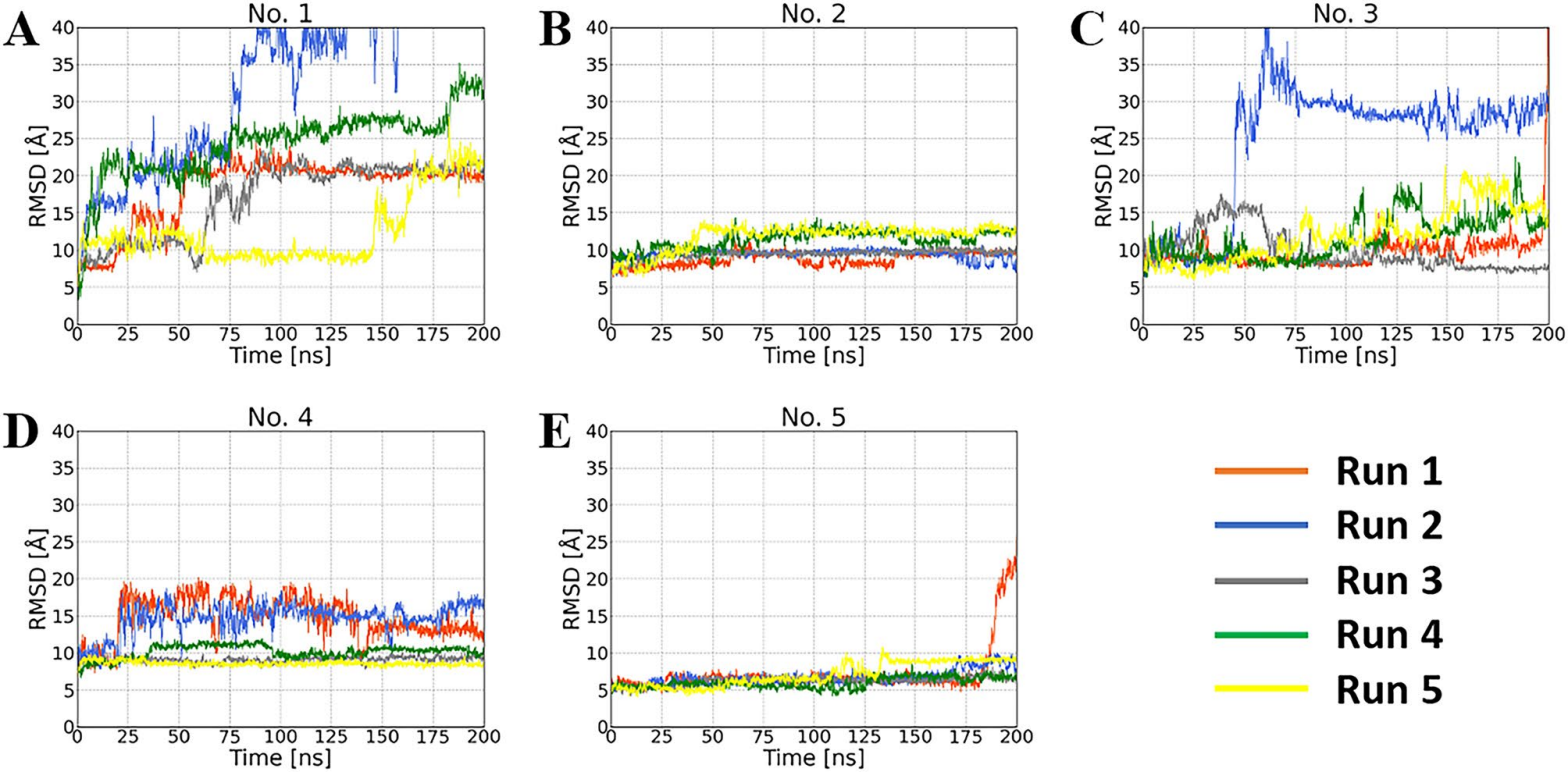
RMSD results of each MD simulation run for each complex structure. (**A**)–(**E**) RMSD calculation based on the heavy atom of the inhibitor in the initial step of the production run. The orange, blue, gray, green, and yellow lines represent the RMSD of MD simulation runs 1, 2, 3, 4, and 5, respectively.

**Figure 4:**
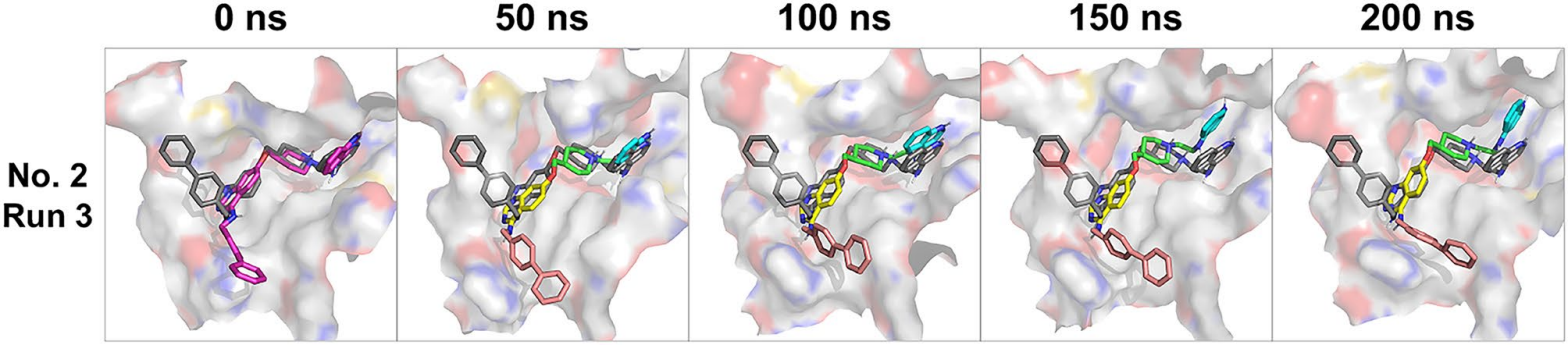
MD models of binding poses with the DNMT3A-selective inhibitor in No. 2. The trajectories of run 3 were used as the representative structure for each docking pose of No. 2. The initial structure of the production run is shown at 0 ns. The binding pose is shown as grayed lines and the docking pose of No. 5 is shown as magenta lines. The four chemical groups of the inhibitor in the model are indicated by different colors (see Fig. 1).

Figure S1 shows RMSD of the C_α_ atom in the protein. All RMSD values of the C_α_ atom were lower than 4 Å, indicating that no arbitrary conformational change occurred in the simulations. The RMSD of the heavy atoms in the inhibitor is shown in Fig. 3. The RMSD values of structure Nos. 1, 3, and 4 were higher than those of the other docking poses (Fig. 3A,C,D). An RMSD greater than 15 Å indicates that the pose of the inhibitor has high fluctuation and significantly differs from the initial structure. Most trajectories of Nos. 2 and 5 had lower fluctuations in RMSD compared with those of the other poses, although the RMSD of run 1 in No. 5 diverged after 175 ns (Fig. 3B,E). These results suggest that the poses of Nos. 2 and 5 maintained binding to DNMT3A. For the inflexible complex structures of Nos. 2 and 5, stability was analyzed by performing binding free energy calculations using the MM/PBSA method. Table 2 shows the results of the binding free energy calculation for each production run for Nos. 2 and 5. Averaged Δ*G*_bind_ of Nos. 2 and 5 were – 28.95 kcal/mol and – 23.96 kcal/mol, respectively. Considering the standard deviation in each run, the binding free energies showed little difference between these poses; the binding poses of the last MD simulations were comparable (Figs. 4, S2, S3). In particular, while the biphenyl group of the inhibitor of Nos. 2 and 5 were located opposite to the initial structure, they were oriented in the same direction in the production run. These results suggest that binding poses of Nos. 2 and 5 are the most inflexible and stable. In particular, the biphenyl group of the inhibitor was stably positioned, as in No. 5.

**Table 2:** Binding free energy for each MD simulation run in Nos. 2 and 5.

Docking rank		Δ*E*_bonded_ ± SD [kcal/mol]	Δ*E*_nonbonded_ ± SD [kcal/mol]	Δ*G*_polar_ ± SD [kcal/mol]	Δ*G*_nonpolar_ ± SD [kcal/mol]	Δ*G*_bind_ ± SD [kcal/mol]
No. 2	Run 1	– 69.75 ± 6.90	99.76 ± 17.89	–51.97 ± 15.22	– 6.08 ± 0.36	– 28.04 ± 5.30
	Run 2	– 76.52 ± 3.57	75.78 ± 16.13	–24.04 ± 13.61	– 6.35 ± 0.24	– 31.13 ± 1.45
	Run 3	– 69.84 ± 3.99	91.10 ± 18.08	–46.53 ± 15.91	– 6.24 ± 0.18	– 31.52 ± 4.72
	Run 4	– 45.43 ± 4.92	76.32 ± 36.85	–55.15 ± 35.33	– 4.60 ± 0.62	– 28.85 ± 5.50
	Run 5	– 48.97 ± 4.13	207.57 ± 35.72	–178.99 ± 32.40	– 4.81 ± 0.47	– 25.20 ± 3.07
No. 5	Run 1	– 57.11 ± 11.00	153.44 ± 55.79	–105.12 ± 48.18	– 5.58 ± 0.93	– 14.37 ± 8.45
	Run 2	– 62.29 ± 9.54	133.23 ± 32.56	–92.75 ± 39.69	– 5.66 ± 0.75	– 27.48 ± 5.44
	Run 3	– 63.73 ± 4.02	164.83 ± 19.60	–114.37 ± 17.55	– 6.40 ± 0.31	– 19.67 ± 5.37
	Run 4	– 63.91 ± 4.92	97.48 ± 26.78	–54.71 ± 26.33	– 6.08 ± 0.40	– 27.22 ± 5.59
	Run 5	– 49.90 ± 6.91	97.98 ± 76.71	–74.21 ± 69.49	– 4.91 ± 0.61	– 31.04 ± 7.46

### Prediction of residues responsible for the selectivity of the inhibitor

**Figure 5:**
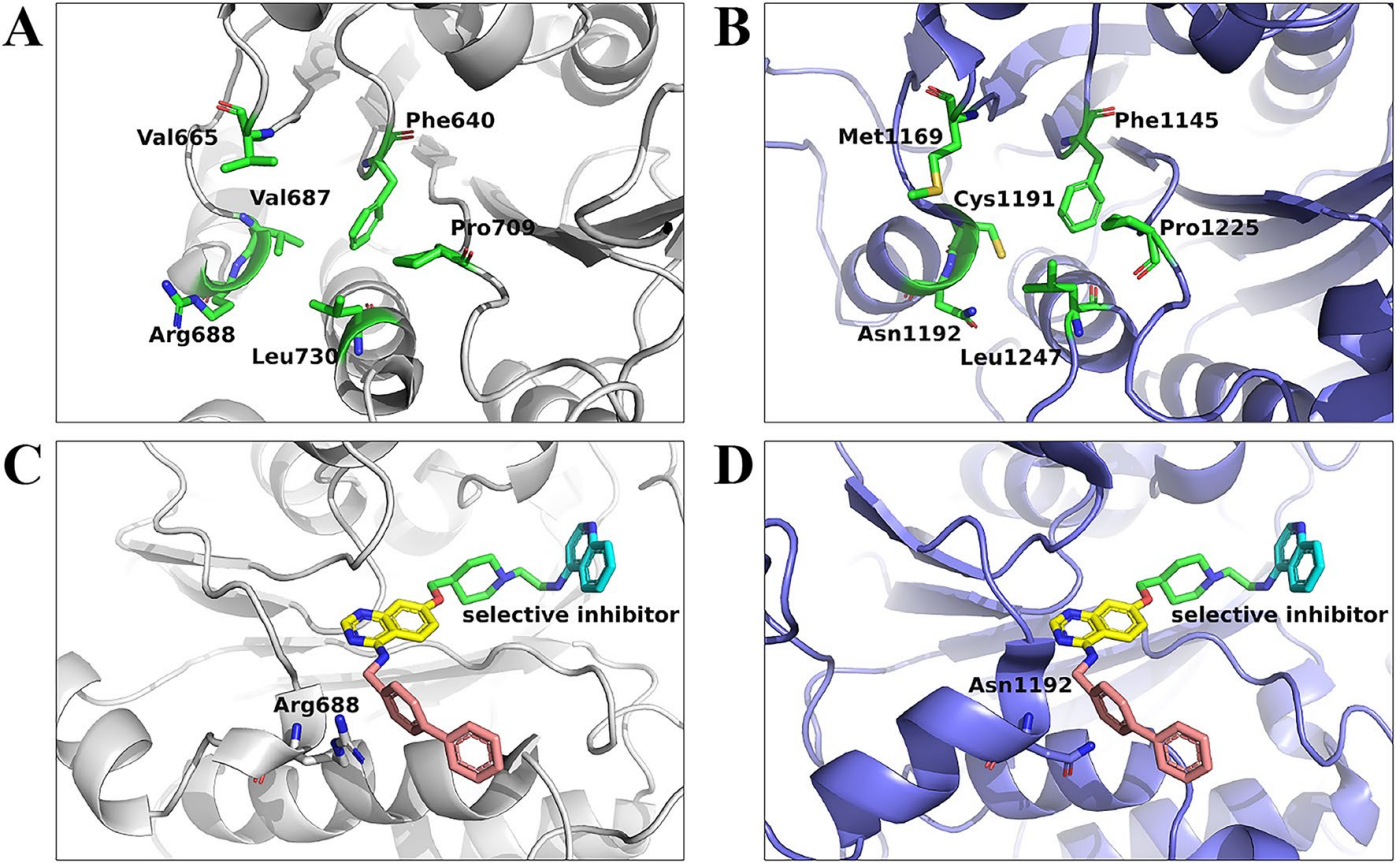
Binding site comparison between DNMT3A and DNMT1. (**A**) 3D positions of the six residues in DNMT3A with high contributions to binding affinity. (**B**) 3D positions of the six residues in DNMT1. (**C**) Structure of the DNMT3A–inhibitor complex. Amino acid residues affecting the selectivity of the inhibitor are shown. The representative structure was selected from a cluster from 500 snapshots of the inhibitor’s conformation recorded at the last 100 ns in five MD simulation runs. (**D**) Representative structure of the inhibitor in complex with DNMT1 based on the structural alignment of DNMT1 and DNMT3A.

For the binding pose of No. 5, which showed little difference between the initial structure and MD snapshots, we analyzed the binding free energy contribution of the amino acid residues of DNMT3A. Figure S4 shows the energy contribution of residues within 6 Å of the selective inhibitor in the initial structure. Arg790 and Arg792 had high decomposition values of 2.121 and 3.242 kcal/mol, respectively. These residues destabilize the binding between DNMT3A and the selective inhibitor. We identified six residues with decomposition values lower than – 1 kcal/mol (Table 3, Fig. S4), which should have a high contribution to the binding of DNMT3A to the selective inhibitor. The types of these residues were compared to those of the corresponding position in DNMT1, and residues with different side chains between DNMT1 and DNMT3A were identified based on ΔΔ*G*_bind_ (Table 3). The positions of these residues in DNMT3A and DNMT1 are shown in Fig. 5A,B. Although Leu730, Phe640, and Pro709 had low decomposition values, the corresponding residues in DNMT1 are the same type as those in DNMT3A; therefore, these residues were not associated with selectivity. Val665 had the highest contribution to the binding free energy, with a value of –2.437 kcal/mol. The corresponding residue in DNMT1 was Met1169, and the ΔΔ*G*_bind_ of Val665Met was − 4.136 kcal/mol, indicating that Met has a higher affinity than Val for binding. Val687 had the second-highest contribution to the binding free energy, with a value of – 1.441 kcal/mol. The corresponding residue in DNMT1 was Cys1191, and the ΔΔ*G*_bind_ of Val687Cys was 0.044 kcal/mol, indicating that Val687 had little effect on the selectivity of the inhibitor. Arg688 had the third-highest contribution to the binding free energy, with a value of – 1.440 kcal/mol. The corresponding residue in DNMT1 was Asn1192; the ΔΔ*G*_bind_ of Arg688Asn was 8.583 kcal/mol, indicating a decrease in binding affinity associated with the mutation of the Asn residue. This suggests that Arg688 in DNMT3A is the key residue influencing the selectivity of the inhibitor for DNMT3A. Indeed, the biphenyl group of the selective inhibitor was stably positioned around Arg688 in MD simulations (Fig. 5C). Arg has a cationic side chain and has a higher affinity for the aromatic ring than Asn, which has a neutral side chain (Fig. 5D). Therefore, the interaction between Arg688 and the biphenyl group influences the affinity and selectivity between DNMT3A and the inhibitor.

**Table 3:** Δ*G*_bind_ decomposition, structural comparison of DNMT3A and DNMT1, and residue scanning calculation.

DNMT3A residue	Δ*G*_bind_ Decomposition (kcal/mol)	DNMT1 residue	ΔΔ*G*_bind_ (kcal/mol)
Val665	– 2.437 ± 0.323	Met1169	– 4.136
Val687	– 1.441 ± 0.348	Cys1191	0.044
Arg688	– 1.440 ± 0.148	Asn1192	8.583
Leu730	– 1.319 ± 0.263	Leu1247	N.D
Phe640	– 1.023 ± 0.951	Phe1145	N.D
Pro709	– 1.008 ± 0.472	Pro1225	N.D

### Comparison with previous structure–activity relationship study

**Figure 6:**
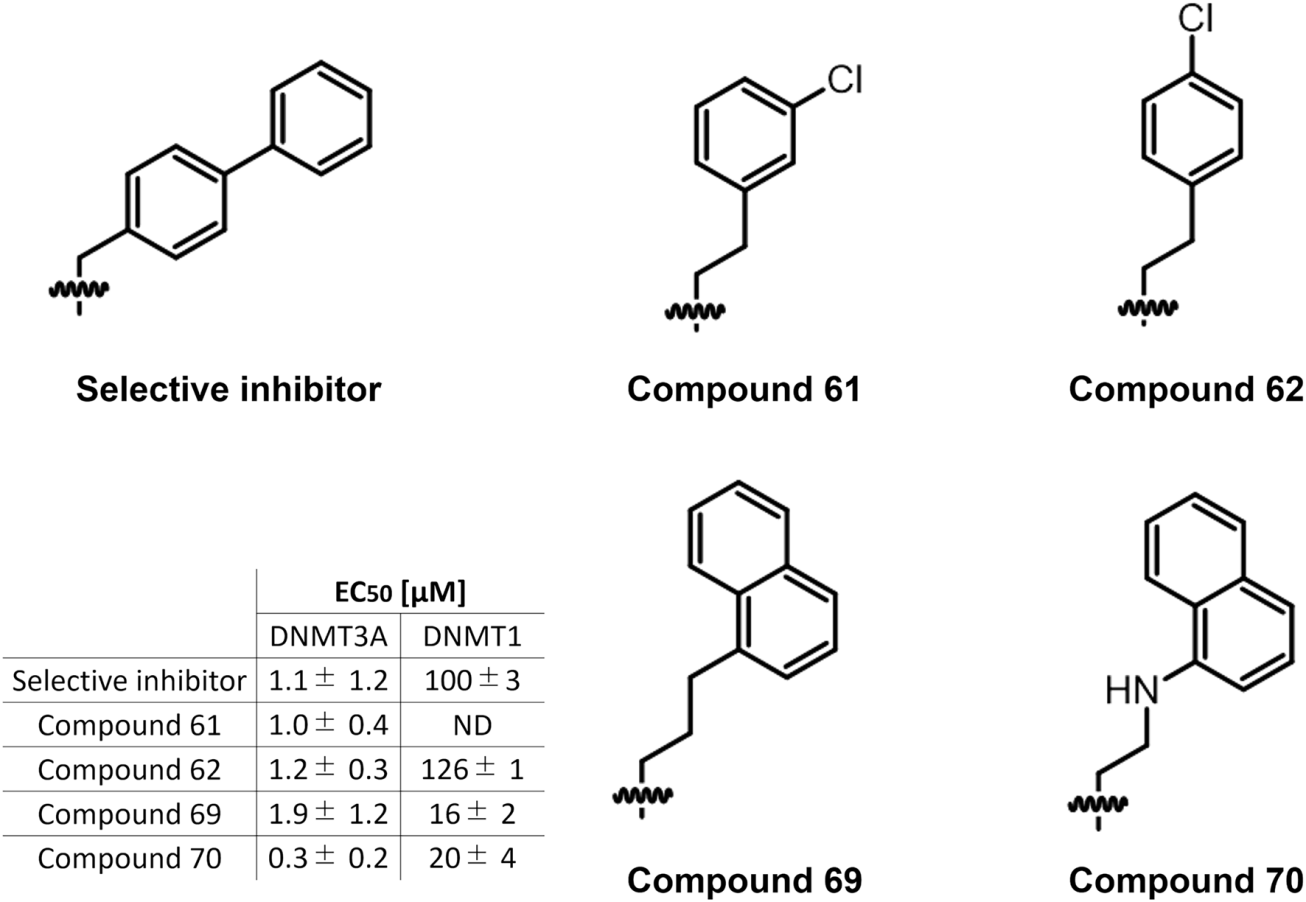
Comparison of substituted groups with high affinity for DNMT3A. EC_50_ values of compounds are referred to the previous study by Halby et al.^20^. Each compound was shown using ChemSketch.

As shown above, Arg688 in DNMT3A may be the key residue influencing the selectivity of the inhibitor toward DNMT3A. In the modeled complex structure, the biphenyl group of the selective inhibitor interacts with Arg688. Halby et al.^20^ identified several other compounds with high affinity to DNMT3A, in addition to the selective inhibitor utilized in our study. For example, EC_50_ values for DNMT3A of compounds 61, 62, 69, and 70 were 1.0 ± 0.4, 1.2 ± 0.3, 1.9 ± 1.2, and 0.3 ± 0.2 μM, respectively (Fig. 6). However, the DNMT3A selectivity of these compounds is insufficient, as the folding values are N.D., 21-, 8-, and 66-fold, respectively. We hypothesized that the insufficient selectivity of these compounds is due to the flexibility of these substituted groups. While the inhibitor selected for our study has only one carbon atom between the nitrogen atom in quinazoline group and the aromatic ring, other compounds have two or three atoms between these functional groups. This difference affects the flexibility of these substructures. Flexible substructures would stably place at the hydrophobic site around Leu730, avoiding being exposed to solvents. Leu730 is conserved in the DNMT1 and 3A, as shown in Table 3. Consequently, compounds with flexible substituted groups maintain high affinity for DNMT3A and potentially reduce DNMT3A selectivity. In contrast, the selective inhibitor chosen for our study has a relatively rigid substructure and can interact with Arg688, thereby exposing it to solvents. These structural insights into the relationships between substructure flexibility and DNMT3A selectivity would be beneficial for the rational design of new DNMT3A selective inhibitors.

## Conclusions

We predicted the inhibitor–DNMT3A complex structure, and the interactions and residues associated with the selectivity of the inhibitor for DNMT3A. Docking and MD simulations predicted that complex structure No. 5 had an inflexible RMSD and stable binding free energy. Structure No. 2 also showed inflexible RMSD and stable binding free energy, and the binding poses after MD simulations were similar to those of No. 5. Structural alignment analysis with known DNMT3A-containing substrates suggested that complex structures, such as Nos. 2 and 5, support the compound design concept of Halby et al.^20^ Amino acid decomposition analysis results showed that Arg688 contributed to the binding between DNMT3A and the inhibitor, which was supported by structural alignment and delta affinity analyses with DNMT1. Our findings using MD simulations could inform drug optimization procedures and support the development of new DNMT-selective inhibitors.

### Supplementary Information

Supplementary Figures.

## Data Availability

All data generated and analyzed during the current study is available from the first and corresponding author on reasonable request.
