# Supplementary material for: Prediction of the binding mechanism of a selective DNA methyltransferase 3A inhibitor by molecular simulation
Source: Sci Rep. 2024 Jun 12;14:13508. doi: 10.1038/s41598-024-64236-9 (PMC11169543; doi:10.1038/s41598-024-64236-9)
Supplement: Supplementary file 1 — Supplementary Figures. [file 41598_2024_64236_MOESM1_ESM.docx]

**Supplementary Materials**

**Prediction of the Binding Mechanism of a Selective DNA Methyltransferase 3A Inhibitor by Molecular Simulation**

Genki Kudo, Takumi Hirao, Ryuhei Harada, Yasuteru Shigeta, Takatsugu Hirokawa, and Ryunosuke Yoshino


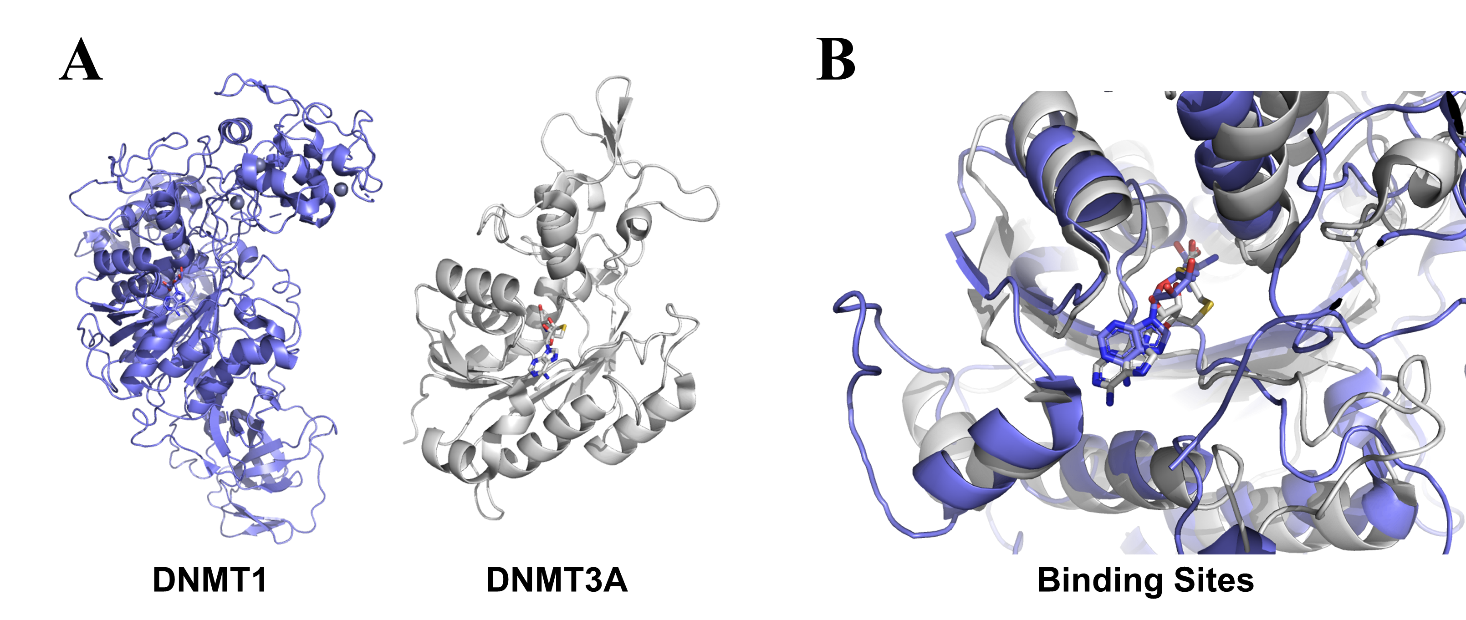


Fig. S1. **Structural comparison between DNMT family proteins.** (A) Entire structure of DNMTs. DNMT1 and 3A are shown as purple and gray cartoon models, respectively. (B) Comparison of binding sites. DNMT1 and 3A are aligned along their backbones.


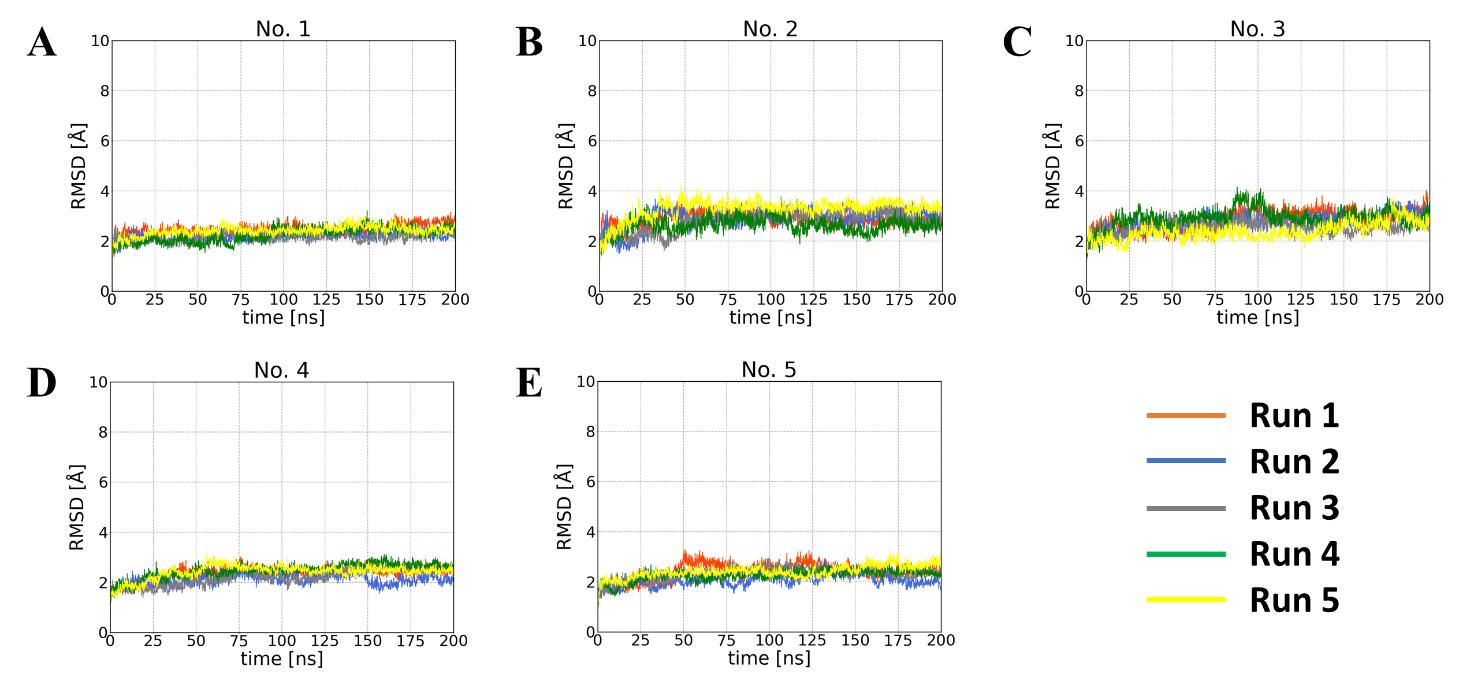


Fig. S2. **RMSD of the protein Cα atom in DNMT3A.** RMSD calculation was based on DNMT3A atoms in the initial step of the production run.


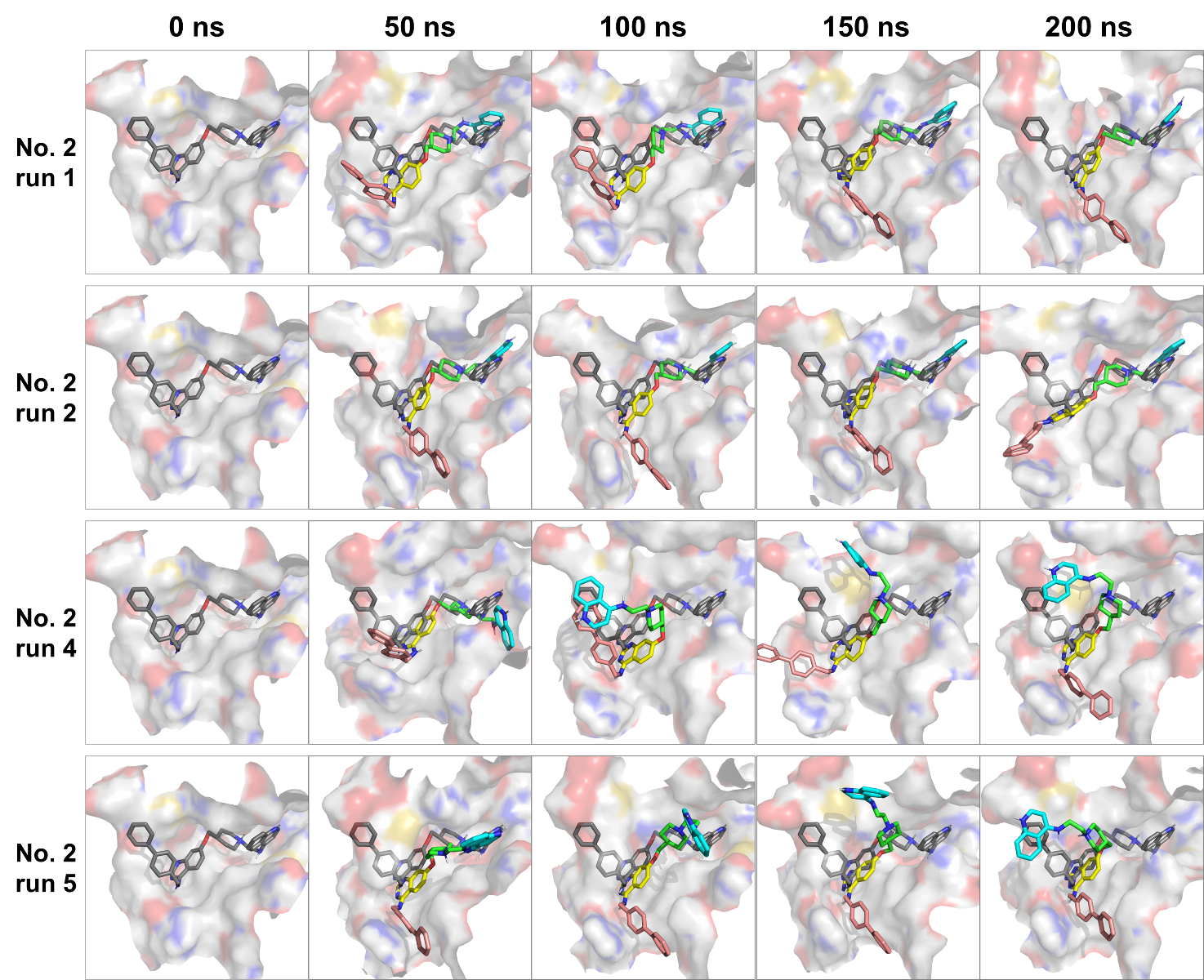
Fig. S3. **MD models of the selective inhibitor in No. 2.**


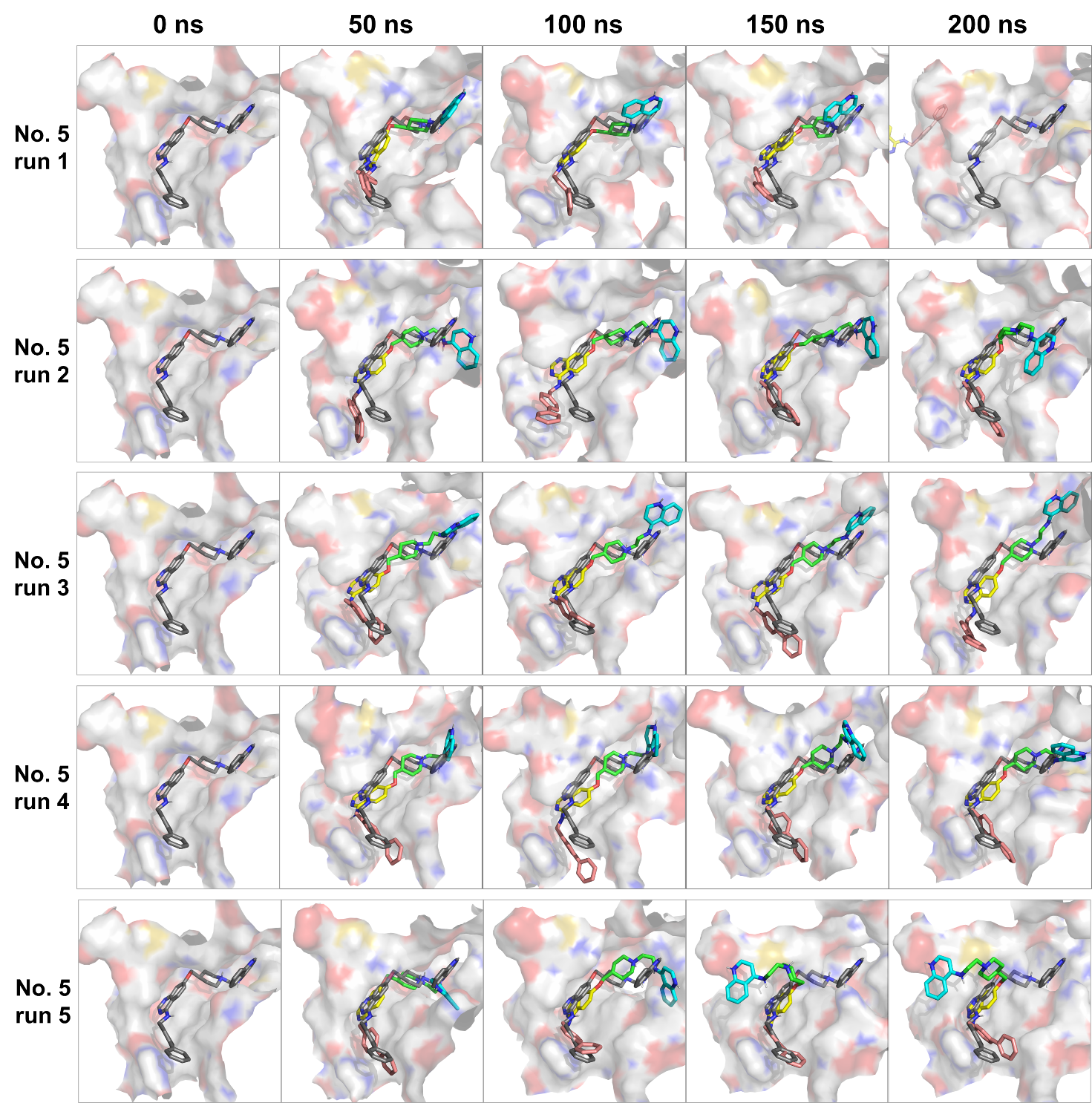


Fig. S4. **MD models of the selective inhibitor in No. 5.**


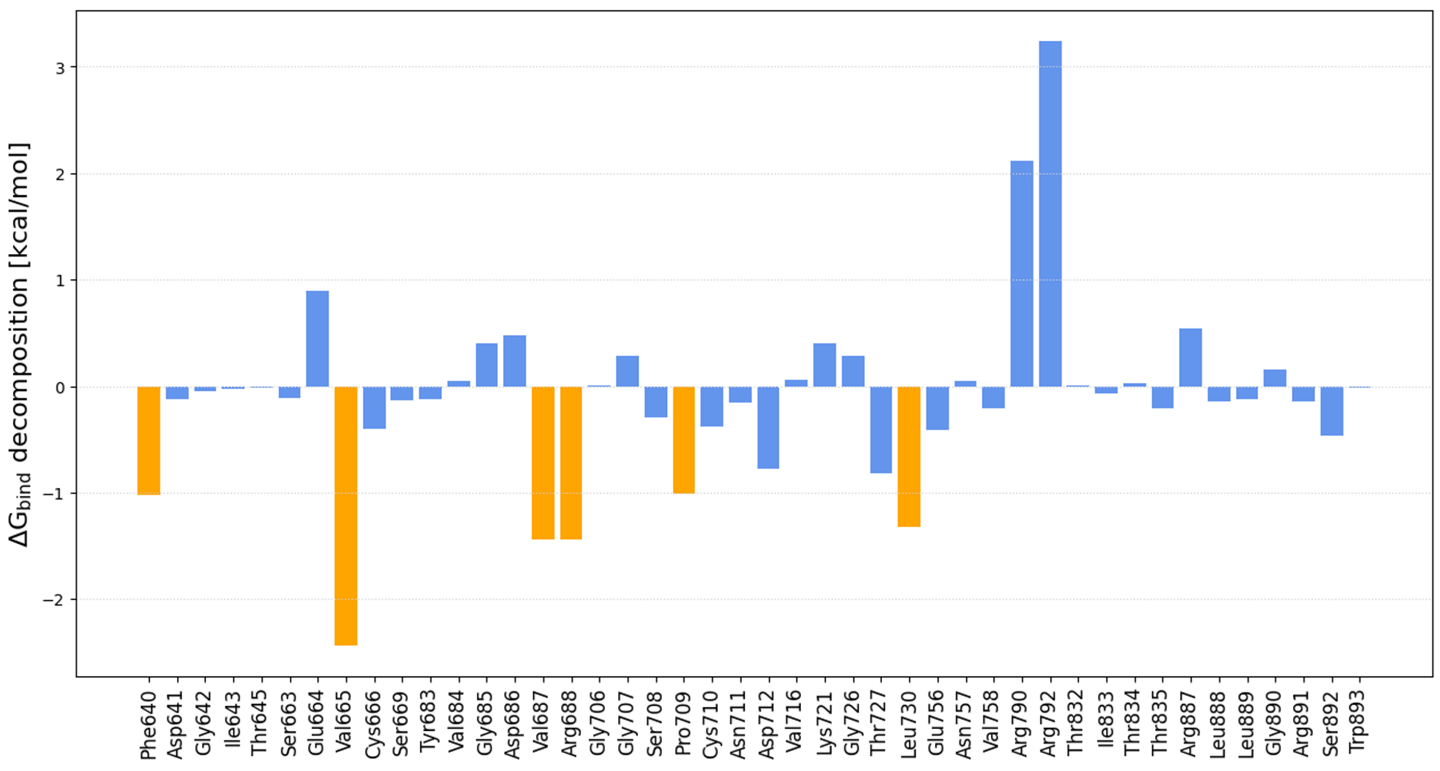


Fig. S5. **ΔG_bind_ decomposition of the residue within 6 Å of the inhibitor.**
